# Ventral cochlear nucleus bushy cells encode hyperacusis in guinea pigs

**DOI:** 10.1038/s41598-020-77754-z

**Published:** 2020-11-26

**Authors:** David T. Martel, Susan E. Shore

**Affiliations:** 1 Kresge Hearing Research Inst., Department of Otolaryngology, University of Michigan, 1100 W. Medical Center Drive, Ann Arbor, MI 48109 USA; 2 Department of Biomedical Engineering, University of Michigan, Ann Arbor, MI 48109 USA; 3 Department of Molecular and Integrative Physiology, University of Michigan, Ann Arbor, MI 48109 USA

**Keywords:** Neuroscience, Diseases of the nervous system

## Abstract

Psychophysical studies characterize hyperacusis as increased loudness growth over a wide-frequency range, decreased tolerance to loud sounds and reduced behavioral reaction time latencies to high-intensity sounds. While commonly associated with hearing loss, hyperacusis can also occur without hearing loss, implicating the central nervous system in the generation of hyperacusis. Previous studies suggest that ventral cochlear nucleus bushy cells may be putative neural contributors to hyperacusis. Compared to other ventral cochlear nucleus output neurons, bushy cells show high firing rates as well as lower and less variable first-spike latencies at suprathreshold intensities. Following cochlear damage, bushy cells show increased spontaneous firing rates across a wide-frequency range, suggesting that they might also show increased sound-evoked responses and reduced latencies to higher-intensity sounds. However, no studies have examined bushy cells in relationship to hyperacusis. Herein, we test the hypothesis that bushy cells may contribute to the neural basis of hyperacusis by employing noise-overexposure and single-unit electrophysiology. We find that bushy cells exhibit hyperacusis-like neural firing patterns, which are comprised of enhanced sound-driven firing rates, reduced first-spike latencies and wideband increases in excitability.

## Introduction

Psychophysical studies characterize hyperacusis as increased loudness growth^1^ over a wide frequency band^2–4^, reduced behavioral response latencies^5^ and decreased tolerance to loud sounds^3^.

Neurons that contribute to hyperacusis should show hyperexcitable firing patterns that reflect the psychophysical characteristics of hyperacusis^6,7^. First, neurons contributing to hyperacusis should show increased firing rates at high intensities. Second, firing rate enhancements should be not restricted to a frequency region, consistent with the wideband nature of hyperacusis. Third, first-spike latencies should be shorter at high intensities, as spike latency reflects the faster reaction times seen in hyperacusis. Fourth, neural responses to sound are expected to be more synchronous, reflecting increased perceptual binding of stimuli.

Previous studies suggest that bushy cells of the ventral cochlear nucleus (VCN) exhibit hyperacusis-like neural activity following noise-overexposure^8,9^. In rodents and other mammalian species, compared to other VCN output neurons, bushy cells show increased phase-locking, a form of neural synchrony, at suprathreshold intensities^10–12^. Moreover, bushy cells show lower and less variable first-spike latencies than other VCN output neurons^10,13,14^ across multiple species^11,12,15^. Following cochlear damage, bushy cells show increased spontaneous firing rates (SFR) across a wide frequency range compared to other CN cell types^16,17^. Following noise-overexposure and putatively in hyperacusis, we predict that bushy cells will show increased sound evoked responses and reduced latencies to higher-intensity sounds. However, no studies have examined bushy cell firing patterns as a function of suprathreshold sound intensity after noise damage with respect to hyperacusis.

While few studies have examined the neural basis of hyperacusis, many studies have examined the neural basis of tinnitus, or phantom sound perception^18–20^. Tinnitus is frequently co-morbid with hyperacusis^4,21^. Unlike hyperacusis, tinnitus occurs in silence and is spectrally similar to hearing loss profiles^22^. Previous studies show that principle output neurons of the dorsal cochlear nucleus (DCN), the fusiform cells, exhibit narrowband increases in SFR and cross-unit spontaneous synchrony to form a neural signature of tinnitus^23,24^. As tinnitus and hyperacusis are frequently co-morbid, it is important to discover whether fusiform cells have a role in hyperacusis, or if bushy cells have a role in tinnitus.

Herein, we hypothesize that VCN bushy cells exhibit hyperacusis-like neural firing patterns that are independent of the DCN-fusiform-cell neural signature of tinnitus. To test this hypothesis, we employ noise-overexposure and single-unit electrophysiology to relate bushy-cell firing patterns to hyperacusis. In noise-overexposed animals, bushy cells exhibit hyperacusis-like neural firing patterns, consisting of 1) increased firing rates, 2) reduced and less variable first-spike latencies, and 3) increases in sound-evoked cross-unit synchrony as a function of intensity across a wide range of frequencies. Furthermore, we compare the hyperacusis-like neural firing patterns seen in bushy cells to the previously established neural signature of tinnitus from fusiform cells. We show that unlike fusiform cells, bushy cells do not show an association between SFR, synchrony and behavioral measures of tinnitus. Rather, bushy cells exhibit enhanced responses as a function of intensity across a wide best-frequency band, consistent with the psychophysics of hyperacusis.

## Results

### Noise-overexposure produces temporary threshold shifts

**Figure 1:**
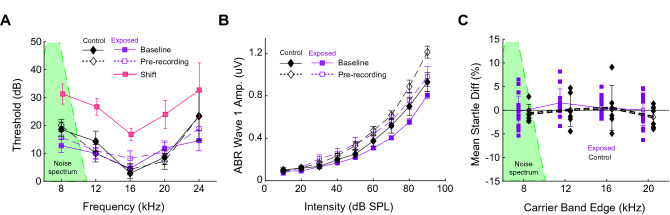
Noise-overexposure results in temporary threshold shifts. (**A**) Following noise-overexposure (spectrum: green triangle), hearing thresholds for exposed animals were elevated immediately post-exposure (filled pink squares) compared to baseline (filled symbols with solid lines) and prior to surgery (open symbols with dashed lines). Hearing thresholds at baseline and prior to single-unit recordings were not significantly different between noise-exposed animals (purple squares) or non-exposed controls (black diamonds). (**B**) ABR W1 amplitude-intensity functions (AIFs) for noise-exposed (purple) and control (black) guinea pigs at baseline (filled symbols with solid lines) and pre-recording (open symbols with dashed lines). (**C**) Mean percent-change in startle amplitude from baseline to post-exposure for each GPIAS carrier band for noise-exposed (purple squares) and control (black diamonds) animals. Data shown are mean + /-SEM.

While hearing loss is the most common factor associated with hyperacusis, it is not essential for its production^4^. Thus, to induce hyperacusis while maintaining normal cochlear function, guinea pigs were noise-overexposed twice in a temporary-threshold shift induction paradigm (see "Methods", Fig. S1 for further detail) (Fig. 1A)^24^. Noise-exposed guinea pigs demonstrated an average threshold shift of 15.9 ± 1.13 dB. Consistent with our previous studies utilizing the same noise-overexposure paradigm, ABR thresholds were not significantly different pre-recording compared to baseline (two-way ANOVA; *p*(group × time) = 0.14). ABR wave 1 (W1) amplitude-intensity functions (AIFs), which are used to estimate cochlear function, were calculated. Noise-overexposed animals showed no significant reduction in ABR W1 amplitude compared to controls across all frequencies and time points (Fig. 1B; two-way ANOVA; F = 2.67; *p* = 0.087), although there was a trend for W1 amplitudes to be smaller in noise-overexposed animals. Startle amplitudes showed no significant differences in noise-exposed animals compared to controls (Fig. 1C; two-way ANOVA; F = 1.04, *p* = 0.38), although a trend for larger startle amplitudes was seen in noise-exposed animals.

### Ventral cochlear nucleus bushy cells exhibit hyperacusis-like firing patterns after noise-exposure

**Figure 2:**
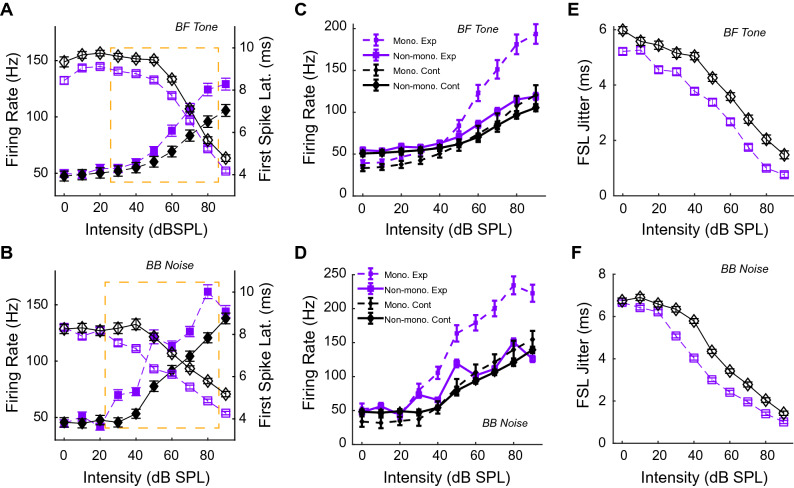
Bushy cells in noise-exposed animals show hyperacusis-like firing patterns. RIFs (filled symbols; left axis) and LIFs (open symbols; right axis) from bushy cells in noise-exposed animals (purple squares with dashed lines) and non-exposed controls (black diamonds with solid lines) in response to (**A**) tones at unit BF and (**B**) broadband noise. Group suprathreshold intensity range indicated by dashed orange boxes. Units were classified as monotonic (dashed-lines) or non-monotonic (solid-lines) from noise-exposed animals (purple) or controls (black) for (**C**) tones at unit BF and (**D**) Broadband noise. FSL jitter, measured as the standard deviation of the FSL, is shown for (**E**) BF tones and for (**F**) broadband noise. Data shown are mean + /-SEM.

Hyperacusis is characterized by enhanced loudness growth at suprathreshold intensities for multiple frequencies^1,25^. Thus, we hypothesized that neurons contributing to hyperacusis would show greater firing rates at higher sound intensities and that these would occur over a wide range of BFs. To test this hypothesis, we recorded from putative VCN bushy cells (n = 1111) across a wide range of BFs (see "Methods" for further detail). Bushy-cells typically show either primary-like or primary-like-with-notch responses to tones at BF^26^. No significant differences were seen between primary-like and primary-like-with-notch units in BF (Student’s t-test; *p* = 0.0531), threshold (two-way ANOVA; *p*(mean × freq) = 0.16) or SFR (two-way ANOVA; *p*(mean × freq) = 0.0531). Thus, data from both unit types were pooled for remaining analyses. We then measured bushy-cell RIFs to BF tone and broadband noise. Noise-exposed animals showed significantly greater RIF slopes at higher sound levels (inset dashed orange boxes) compared to controls for BF tones (Fig. 2A; filled symbols; *p* = 3.56e-4; two-sample t-test) and broadband noise (Fig. 2B; filled symbols; *p* = 6.2e-3; two-sample t-test), consistent with an expected enhanced suprathreshold loudness growth seen in hyperacusis^25,27^. Human psychoacoustic studies also demonstrate reduced reaction times in subjects with hyperacusis^5,28^, which are thought to arise from a hyperexcitable auditory pathway. To assess neural excitability, we analyzed bushy-cell first-spike latencies (FSL) as a function of intensity. Bushy cells in noise-exposed animals showed steeper FSL slopes as a function of intensity for BF tones at suprathreshold intensities compared to control animals (Fig. 2A; open symbols; two-sample t-test; *p* < 0.0081). The decrease in FSL was even more pronounced in response to broadband noise (Fig. 2B; open symbols; two-sample t-test; *p* < 0.0019). Moreover, bushy-cell RIFs can show non-monotonicity at high intensities, partially arising from wideband inhibition provided by VCN D-stellate neurons^29–31^. Noise-overexposure reduces inhibition in other cochlear nucleus neurons^8^. Thus, a loss of inhibition of bushy-cells due to noise-overexposure might result in more monotonic RIFs. RIFs were tested for monotonicity^32^, which was quantified by calculating the non-Monotonicity Fraction (nMF). Compared to control animals, noise-exposed animals had a significantly greater proportion of monotonic RIFs (BF-tones see Fig. S2A; *p* = 0.0154, chi-stat = 5.86; BBN RIFs see Fig. S2B; *p* = 2.31e-12, chi-stat = 49.2). The greater fraction of monotonic RIFs in noise-exposed animals is reflected in steeper monotonic RIF slopes compared to monotonic RIFs from control animals for BF-tone (Fig. 2C) and BBN noise (Fig. 2D). Neural excitability can also be assessed by spiking reliability, or spike jitter, in response to a stimulus. More excitable neurons should more reliably produce spikes at stimulus onset compared to less excitable neurons. We quantified the bushy cell spike jitter by measuring FSL standard deviation. Bushy cells in noise-exposed animals exhibited reduced FSL jitter to BF tones (Fig. 2E; two-sample t-test, *p* < 5.34e-5) and to broadband noise (Fig. 2F; two-sample t-test, *p* < 5.46e-23) compared to control animals.

**Figure 3:**
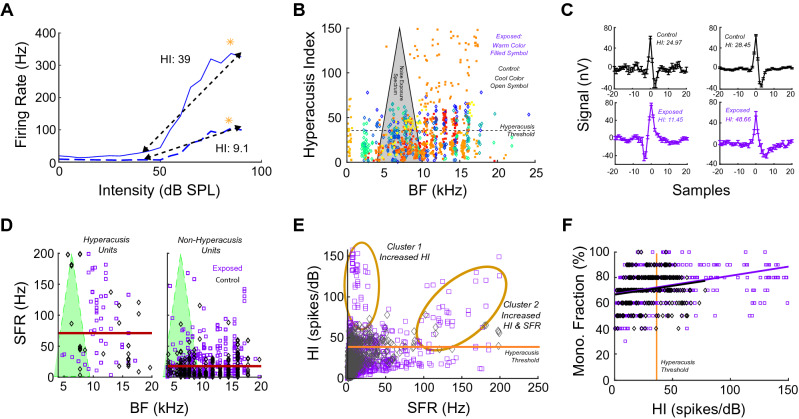
Noise-exposed animals show elevated Hyperacusis Indices (HI) compared to controls. (**A**) HI, shown for two example RIFs, was calculated from the geometric mean of: 1) the average RIF slope from 40–90 dB (dashed black line) and 2) peak firing rates over the same intensity interval (orange star). (**B**) HI vs BF for noise-exposed animals (warm color, filled, square symbols) and non-exposed controls (cool color, open, diamond symbols) relative to the noise-exposure spectrum (black triangle with grey background). Hyperacusis threshold shown as dashed black line; each color denotes data the same animal. (**C**) Threshold-triggered mean spike waveforms were identified from single units with a range of HI values (inset text) and from noise-exposure status (upper panels: controls in black; lower panels: noise-exposed in purple). Single-unit spike waveforms not distinguished by HI or noise-exposure status. For each waveform, N >  = 20 spike snippets included. Data shown are mean + /-SEM. (**D**) Left panel: SFR by BF for Hyperacusis units, with distribution mean (orange line). Right panel: SFR by BF for non-Hyperacusis units, with distribution mean (orange line). (**E**) HI versus SFR, with Hyperacusis-unit clusters indicated by orange ellipses. Hyperacusis threshold line shown in orange. (**F**) Monotonic fraction of a unit (100-nMF%) plotted with the unit’s HI. Hyperacusis Threshold (HI = 35.5) shown (solid, vertical orange line).

Next, we examined the relationship between bushy-cell evoked firing rate patterns and hyperacusis characteristics by constructing a neural “Hyperacusis Index” (HI) for each neuron. The HI is equal to the geometric mean of (1) the RIF slope in response to sounds from 40 dB SPL to 90 dB SPL (dashed black line in Fig. 3A) and (2) the peak firing rate of the RIF (orange stars in Fig. 3A). Neither primary-like nor primary-like-with-notch units exhibited significant differences in HI (two-way ANOVA; *p*(mean × freq) = 0.75), and so data from both unit types were pooled for the following analyses. To determine whether an animal had hyperacusis, an unsupervised cluster analysis was performed by fitting HI with a two-member Gaussian-mixture model. A unit was defined as a *Hyperacusis Unit* if the probability of assignment to the elevated HI cluster was greater than 0.5, which corresponded to HI = 35.5 (Hyperacusis threshold) (Fig. S2C). 25.5% of units were classified as *Hyperacusis Units*, while the remaining 74.8% of units were *Non-Hyperacusis Units*. Noise-exposed animals had more units with HIs above the Hyperacusis threshold (n = 189) compared to non-exposed control animals (n = 94; dashed black line in Fig. 3B). *Hyperacusis Units* were found across a wide range of frequencies in individual animals (BF range: 0.32–24.3 kHz), consistent with the reported wideband characteristics of hyperacusis^1,2^ (Fig. 3B). To ensure that *Hyperacusis Units* were composed only of single-units, threshold-triggered mean spike waveforms were assessed. Spike waveforms did not show any evident differences across a range of HIs or animal’s noise-exposure status (sample spike waveforms shown in Fig. 3C). The *Hyperacusis Units* also showed significantly elevated SFR across all frequencies (left panel in Fig. 3D; two-way ANOVA; *p*(SFR) = 5.38e-87, *p*(freq) = 0.26; mean indicated by dark red line) compared to the *non-Hyperacusis Units* (right panel in Fig. 3D; mean indicated by dark red line). A Gaussian mixed model fit to HI and SFR data revealed that the *Hyperacusis Units* could be separated into two clusters, one of which demonstrated elevated HI (cluster 1 in Fig. 3E), while the second showed elevated HI and elevated SFR (cluster 2 in Fig. 3E).

We found that RIFs in noise-exposed animals were disproportionately monotonic compared to controls animals, which suggested that disinhibition may play a role in hyperacusis-like firing in the bushy cells. To relate disinhibition to HI, each unit’s Monotonicity Fraction (MF) was calculated (100-nMF) and plotted with HI (Fig. 3F). While HI and MF were strongly correlated for both exposed (Pearson’s linear correlation; r = 0.34, *p* = 3.32e-18) and control (r = 0.19, *p* = 8.14e-4) animals, the exposed animal MF-HI correlation was nearly twice as large.

### Hyperacusis and tinnitus have distinct neural substrates

Tinnitus and hyperacusis are frequently, but not always, co-morbid^2,4^. Since our data suggested that some noise-exposed animals show hyperacusis-like neural firing patterns, we considered that some of the noise-exposed animals might also show neural and behavioral evidence of tinnitus. Prior to single-unit recordings, animals were tested for tinnitus using gap-prepulse inhibition of the acoustic startle (GPIAS)^24,33,34^, in which animals are diagnosed with tinnitus if they exhibit impairments in gap-prepulse detection (see "Methods" for further detail). Ten out of twenty-two noise-exposed animals demonstrated significantly impaired gap-prepulse detection in at least one tested frequency-band, while no control animals demonstrated gap-prepulse impairments at any frequency-band. To control for potential frequency-specific temporal-processing deficits, noise-PPI was also assessed^18^. No animals showed significant deficits in noise-PPI, consistent with previous studies utilizing the same paradigm^23,24,35,36^.

**Figure 4:**
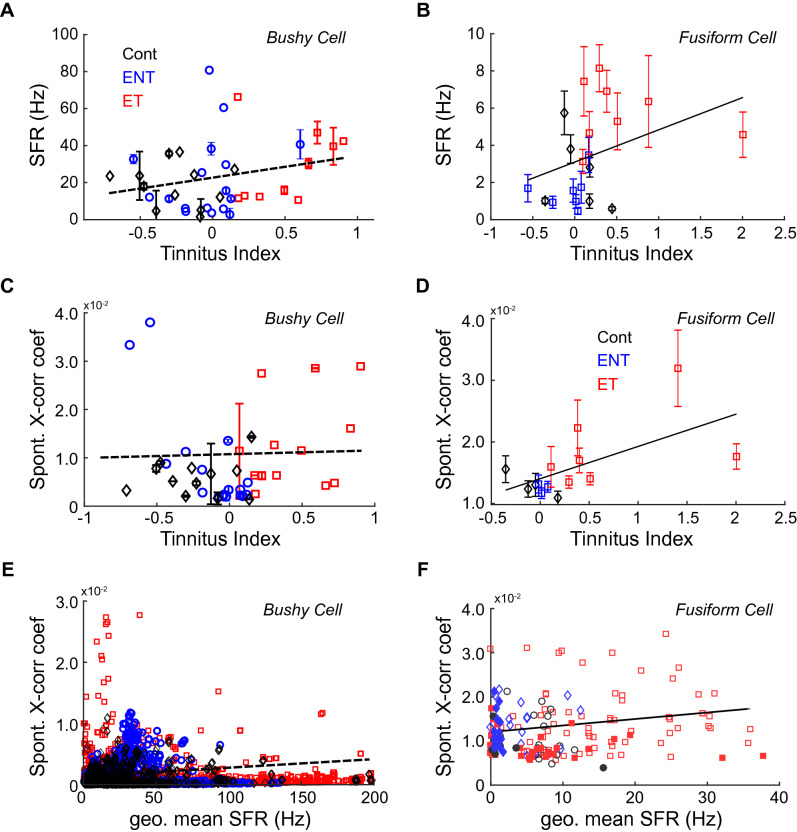
Bushy cell spontaneous firing does not relate to tinnitus behavior compared to fusiform cells. Data colored based on tinnitus-status (exposed-tinnitus are red, exposed-no-tinnitus are blue, and non-exposed controls are black). (**A**) Bushy cell SFR, when binned by BF for each Tinnitus Index (TI) carrier-band, does not significantly correlate with TI (r = 0.06, p = 0.14). (**B**) Fusiform cell SFR strongly correlates with frequency-matched TI values (r = 0.21, p = 1.7e-8). (**C**) Bushy cell cross-unit spontaneous synchrony (Spont. X-corr coef), binned by BF per TI-carrier bands, does not significantly correlate with TI (r = 6.7e-4, p = 0.87). (**D**) Fusiform cell X-corr coef significantly correlate with frequency-matched (TI r = 0.21, p = 0.026). (**E**) Bushy cell X-corr coef weakly correlates with geometric mean SFR of contributing unit-pair (r = 0.049, p = 3.38e-5). (**F**) Fusiform cell X-corr coef strongly correlates with geometric mean SFR in animals with tinnitus (r = 0.21, p = 0.026). Panels B, D and F republished with permission from authors in Wu et al.^24^.

To assess whether bushy-cell spontaneous activity contributed to tinnitus, frequency-specific SFR and cross-unit spontaneous synchrony (X-corr coef), two neural hallmarks of tinnitus^24,37,38^, were examined. We found that mean SFR was increased in bushy cells in tinnitus animals compared to exposed, no-tinnitus animals and controls (exposed, tinnitus = 30.18 Hz (ET, red symbols); exposed, no-tinnitus = 22.42 Hz (ENT, blue symbols); control = 18.89 Hz (CONT, black symbols)). Further, bushy-cell SFR was increased across all BFs (two-way ANOVA; *p*(mean) = 3.04e-2; *p*(freq) = 0.18). Wideband increases in bushy cell spontaneous activity may be more reflective of hyperacusis than tinnitus, which shows BF-restricted increases. To further quantify tinnitus behavior within a frequency band, we computed a tinnitus index (TI), a startle response-based metric to quantify “tinnitus strength” for each animal (see "Methods" for further detail). When binned by BF and TI carrier-band-frequency, bushy-cell SFR did not significantly correlate with the TI (Fig. 4A; Pearson’s correlation; r = 0.06, *p* = 0.14) compared to that of DCN fusiform cells (Fig. 4B; Pearson’s correlation, r = 0.21, *p* = 1.7e-8)^24^. Furthermore, BF-specific increases in cross-unit spontaneous synchrony did not correlate with an animal’s TI (Fig. 4C; Pearson’s correlation; r = 6.7e-4, *p* = 0.87). In contrast, fusiform cell cross-unit spontaneous synchrony highly correlates with TI (Fig. 4D; Pearson’s correlation; r = 0.21, *p* = 0.026). These findings suggest bushy-cell spontaneous activity does not contribute to tinnitus, in contrast to spontaneous activity from fusiform cells.

Previous studies have demonstrated that synchronous activation of neurons contributes to perceptual binding^39,40^. In tinnitus, enhanced cross-unit spontaneous synchrony would thus signal the presence of sound in the absence of sound^18,24,41,42^. However, synchrony measurements should control for baseline spontaneous activity, as more spiking can create more opportunities for correlations. Thus, when correlation coefficients are normalized by the number of spikes in each spike train^24,43^, enhanced correlations between synchrony and SFR indicate that spiketrains are more similar than predicted by chance. While bushy-cell SFR significantly correlated with synchrony in tinnitus animals (Fig. 4E; Pearson’s correlation; r = 0.049, *p* = 3.38e-5), the correlation was four times smaller than the correlation between SFR and synchrony previously shown in fusiform cells (Fig. 4F; Pearson’s correlation; r = 0.21, *p* = 0.026)^24^. These findings suggest that, while bushy cells are more spontaneously active in animals with tinnitus, bushy cells with elevated spontaneous activity do not show cross-unit synchronous firing like that in fusiform cells in animals with tinnitus.

**Figure 5:**
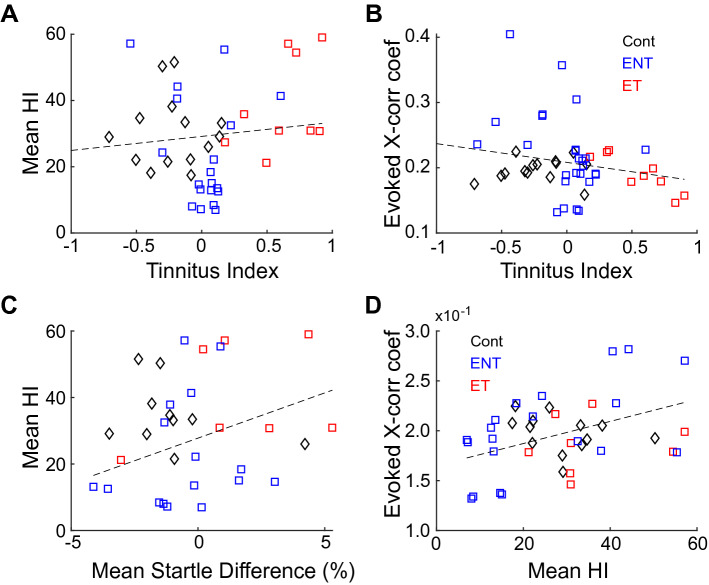
Bushy cell sound-evoked activity relates to hyperacusis and not tinnitus. (**A**) Bushy-cell mean HI, when binned by BF using TI carrier-bands, does not correlate with TI. (**B**) Evoked synchrony, binned by geometric BF of each unit-pair relative to TI carriers, does not significantly correlate with TI. (**C**) Mean HI correlates with the percent change in non-prepulse startle amplitudes. (**D**) Evoked synchrony correlates with mean HI.

To relate tinnitus behavior to hyperacusis in bushy cells, we binned HI by carrier frequencies used in the GPIAS tests. HI in a tinnitus frequency-band did not correlate with the corresponding TI (Fig. 5A; Pearson’s correlation, *p* = 0.43, r = 0.12). Furthermore, evoked cross-unit synchrony, a measure of stimulus binding potentially reflective of hyperacusis, did not correlate with the corresponding frequency-matched TI (Fig. 5B; Pearson’s correlation, r = -0.23, *p* = 0.11). However, HI significantly correlated with the percent change in startle amplitude from baseline to pre-recording (Fig. 5C; Pearson’s correlation, *p* = 0.05, r = 0.35). Further, evoked synchrony correlation coefficients significantly correlated with binned HI (Fig. 5D; Pearson’s correlation, r = 0.43, *p* = 0.021). These findings suggest that bushy-cell evoked activity unlikely contributes to tinnitus, but instead is more consistent with hyperacusis.

**Figure 6:**
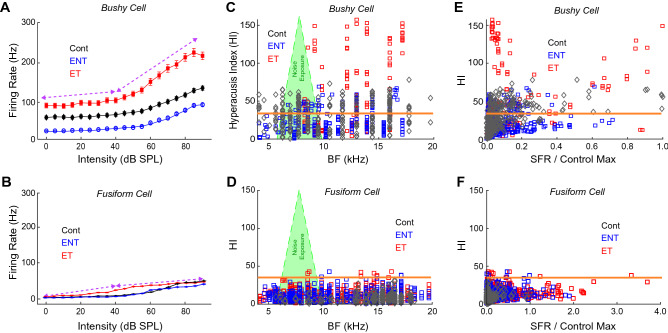
Bushy cell hyperacusis-like firing patterns distinct from fusiform cell measures of hyperacusis. (**A**) Bushy cells in tinnitus animals show suprathreshold RIF slope increases from 40 to 90 dB (dashed purple lines). (**B**) Rate-intensity functions in FCs do not show suprathreshold slope increases in tinnitus animals from 40 to 90 dB (dashed purple lines). (**C**) Elevated HIs occur predominantly, but not exclusively, in noise-exposed animals. Hyperacusis threshold line computed from bushy-cell HI distribution shown in orange. (**D**) HI is not increased in fusiform cells in noise-exposed animals compared to controls or to bushy cells. (**E**) Bushy cell elevated HI-SFR clusters not-exclusively linked to tinnitus-status. (**F**) FCs do not show distinct clusters of elevated HI but do show enhanced SFR. Data in Panels from B, D and F re-analyzed with permission from authors in Wu et al.^24^.

We then asked how bushy-cell evoked activity compared to fusiform-cell evoked-activity. The most striking difference between bushy cells and fusiform cells is the order-of-magnitude greater evoked firing rates in bushy cells compared to fusiform cells, with concurring increases in firing-rate slope. HIs and RIFs from bushy cells and fusiform cells were grouped by tinnitus-status. Bushy-cell RIFs showed greater suprathreshold increases in firing in tinnitus animals compared to non-tinnitus animals and controls (upper purple arrow in Fig. 6A). In contrast, fusiform cell RIFs were not enhanced at suprathreshold intensities (upper purple arrow in Fig. 6B) in exposed, tinnitus animals compared to either exposed, no-tinnitus animals or controls. Moreover, bushy cells in both tinnitus and no-tinnitus animals had HIs above the previously established Hyperacusis threshold (orange line in Fig. 6C), while fusiform cells did not show any distinct elevations of HI over BF in noise-exposed animals compared to controls (two-way ANOVA; *p*(freq) = 0.81, *p*(mean) = 0.31). Furthermore, less than 1% of fusiform-cell HIs were above the bushy-cell Hyperacusis threshold (orange line in Fig. 6D). To verify the lack of HI clustering in fusiform cell data, Gaussian-mixture model fits with different starting centroids did not consistently produce clusters that converged to final values, suggesting that distinct HI clusters are not an intrinsic feature of this dataset. As shown in Fig. 3E, bushy cells show distinct clusters when plotting HI versus SFR. While most of the data points in each cluster were from animals with tinnitus, data from animals without tinnitus (either exposed or control) were also present (Fig. 6E), suggesting that the clusters are not part of a tinnitus signature. In contrast to bushy cells, there were no distinct clusters of HI versus SFR in fusiform cells, which instead showed large increases in SFR compared to control animals (Fig. 6F). These findings reiterate that fusiform-cell firing patterns following noise-overexposure and tinnitus induction are more reflective of tinnitus, as previously shown, and are inconsistent with the hyperacusis-like neural firing patterns found in bushy cells.

## Discussion

In the present study, we showed that VCN bushy cells in noise-overexposed guinea pigs show hyperacusis-like neural firing patterns consisting of steepened rate-intensity functions, reduced and less variable first-spike latencies, and increases in sound-evoked cross-unit synchrony across a wide BF range. Consistent with human studies, in which there is significant co-morbidity between hyperacusis and tinnitus, some putative hyperacusis animals also showed behavioral evidence of tinnitus. We also find that analysis of data from fusiform cells indicated that they do not exhibit hyperacusis-like firing patterns, in contrast to bushy cells.

### Mechanisms underlying enhanced bushy-cell excitability

There are several possible mechanisms by which bushy cells could become more excitable after noise exposure, resulting in hyperacusis-like firing. Bushy cells in rodent and mammalian species receive somatosensory input on their dendrites which can modulate firing sensitivity^36,44^. Following noise-exposure, the anterior VCN receives increased glutamatergic input from non-auditory structures^36^. Increased glutamatergic somatosensory input^45^ could potentially reduce the threshold for bushy cell spiking, resulting in enhanced firing rates and reduced latencies, consistent with the present findings.

In addition to somatosensory input, cholinergic signaling is altered in VCN following noise-exposure^46,47^ indicated by increases in choline acetyltransferase (ChAT) and muscarinic ACh receptors in the CN. These studies propose that cochlear damage triggers homeostatic increases in ACh-mediated excitability to compensate for reduced auditory nerve output. Interestingly, cochlear insult with carboplatin, a ototoxic anticancer drug, was shown to enhance VCN, but not DCN, expression of Growth Associated Protein (GAP) 43^48^. Increases in GAP-43, reflecting axon growth and synaptogenesis, could result in enhanced synchronization of bushy cells through putatively increased collateralization of bushy-cell projections. Increases in VCN neural excitability could also arise through local disinhibition, as measured through reductions in glutamic acid decarboxylase 65 (GAD65) expression^8^, a GABA-terminal marker. In that study, reductions in GAD65 co-occurred with increases in startle amplitude and ABR wave 2:1 amplitude ratio, both measures which are predicted to correlate with hyperacusis.

However, reductions in cochlear output does not need to be permanent to produce increases in CN excitability. Transient reductions in auditory nerve input can also elicit homeostatic increases in CN excitability. Ear plugging can result in increased AMPA in the post-synaptic density (PSD) of bushy cells, making them cells more excitable^49^. This increase is sustained after the ear canal is re-opened. Animals with increased AMPA receptor expression and thicker PSDs also showed enhanced later ABR wave amplitudes compared to controls. ABR waves 2 and 3 are predominantly generated by the synchronous firing of bushy cells while waves 4 and 5 are generated by bushy-cell targets in the auditory pathway^14^, suggesting that the bushy cell pathway becomes more excitable as a result of enhanced AMPA receptor expression. Other studies have shown that following conductive hearing loss, contralateral inputs to the VCN become more excitatory, resulting in increased SFR and enhanced sensitivity to broadband noise^50^. These findings suggest that transient insults to the auditory pathway can have long-lasting changes resulting in a hyperexcitable auditory pathway.

Hyperacusis-like firing patterns could also arise from bushy-cell network changes beyond cellular changes. Bushy cells are part of an electrotonically-coupled network that may allow for the rapid spread of excitation^51,52^. Enhanced network connectivity could allow bushy cells to fire more rapidly in response to sound with increased synchrony and reduced latency, consistent with the present findings. A similar mechanism has been proposed to account for perceived brightness in visual cortex: increases in synchrony of stimulus-evoked firing rates correlated with enhancements in perceived brightness^53,54^.

Bushy cells receive inhibitory interneuron input, including wideband inhibition from VCN D-stellate neurons, and narrowband input from DCN vertical cells^55–58^. In normal-hearing animals, inhibitory input onto bushy cells is essential for maintaining phase-locking in the presence of background noise^59,60^. By transiently raising bushy-cell spike thresholds, out-of-phase subthreshold membrane summation is prevented from eliciting spikes. Disinhibition of D-stellate or vertical-cell input would result in more spikes that are less phase-locked to stimuli, than those influenced by auditory-nerve input, consistent with the present results. Moreover, reduced D-stellate cell input onto bushy cells would also result in enhanced bushy-cell sensitivity to off-BF sounds, consistent with the wideband nature of hyperacusis. Reduced d-stellate input onto the bushy cell is consistent with the reduction in non-monotonicity at suprathreshold intensities in noise-exposed animals compared to controls: sideband inhibition increases with increasing sound bandwidth as well as at higher intensities^61^. Loss of inhibitory input from d-stellate neurons would result in greater bush-cell firing rates at the highest intensities. Moreover, we found that BBN RIFs in exposed animals show far greater loss of non-monotonicity compared to BF-tone RIFs (Fig. S2A, B). BBN RIFs arise from stimuli with greater bandwidth, which implies a greater role for d-stellate neurons in hyperacusis compared to the narrowband vertical cell inhibition. Regardless, future studies should investigate the roles of D-stellate and vertical cells in hyperacusis.

### Co-morbidity of hyperacusis and tinnitus

Tinnitus and hyperacusis are frequently co-morbid, with an average co-incidence rate across studies of 60%^1–3^. Consistent with this observation, we found that a subset of noise-exposed animals shows electrophysiological evidence for both hyperacusis and tinnitus. However, not all hearing loss leads to either tinnitus or hyperacusis; moreover, hyperacusis and tinnitus can occur independently of each other^4^. In the present study, all unique combinations of hyperacusis and tinnitus were seen. We found that 50.8% of *Hyperacusis Units* come from animals with tinnitus (tinnitus-with-hyperacusis), while 27.3% of *non-Hyperacusis Units* were from tinnitus animals (tinnitus-without-hyperacusis). These proportions suggest that tinnitus-with-hyperacusis is the most common condition resulting from our noise-exposure, which is also consistent with human studies, as the average coincidence rate of tinnitus and hyperacusis is greater than 50%. Further, 25.1% of *Hyperacusis Units* came from exposed, non-tinnitus animals (hyperacusis-without-tinnitus), suggesting that some of the non-tinnitus animals might also have hyperacusis.

We found that most *Hyperacusis Units* are found in tinnitus animals. However, bushy-cell HI did not correlate with an animal’s TI when binned with TI-carrier frequencies. These findings suggest that there could instead be a fusiform-cell contribution to tinnitus in animals with bushy-cells showing elevated HI. A reduction in wideband-inhibition from D-stellate neurons could likewise reduce inhibition of the fusiform cell^31,44^, potentially linking hyperacusis arising from bushy cells in the VCN with tinnitus arising from fusiform cells in the DCN. Future studies should perform concurrent bushy-cell and fusiform-cell recordings in the same animals, along with behavioral measures of tinnitus and hyperacusis.

### Behavioral models of hyperacusis

While several behavioral tests for hyperacusis have been proposed^25,28,62–65^, there are no widely-adopted behavioral tests for hyperacusis^66,67^. Current paradigms utilize either enhanced startle amplitudes or reduced reaction times as hyperacusis-measures. As bushy-cells in noise-exposed animals showed both increases in firing rate and reductions in first-spike latency, we propose that a combined measure of both reaction times and startle amplitudes will measure hyperacusis. We predict that animals with hyperacusis will show enhanced startle response amplitude slopes as well as reduced startle reaction time slopes as a function of stimulus intensity, as suggested by the bushy cell RIF enhancements and LIF reductions seen in noise-exposed animals. Further, because bushy cells in noise-overexposed animals show greater absolute RIF and LIF slopes in response to broadband noise than to BF tones, we predict that animals with hyperacusis will show stronger responses to broadband noise, as wideband sounds are perceived to be louder than SPL-equivalent narrowband sounds^68^.

In the present study, noise-exposed animals showed a trend for increases in startle amplitude at several frequency bands. This trend is consistent with another hyperacusis study, which showed that suprathreshold startle enhancements, reflective of hyperacusis, were seen only in response to startle pulses greater than 100 dB^25^, but not in response to the 90 dB SPL stimulus used in the present study. Future studies should measure startle-amplitude-intensity functions, with peak sound outputs greater than utilized in the present study, to assess hyperacusis-behavior.

## Methods

### Ethical treatment of animals

All animal procedures were performed in accordance with protocols established by the National Institutes of Health (Publication 80–23) and approved by the Institutional Animal Care and Use Committee at the University of Michigan.

### Experimental design

N = 29 female pigmented guinea pigs were obtained from the Elm-Hill colony at 2–3 weeks of age. Animals were noise-overexposed using a paradigm previously established in the lab (Fig. S1A). Baseline auditory brainstem responses (ABRs) were measured to establish normal hearing followed by four weeks of behavioral testing. All noise-exposed animals received two noise exposures separated by 4 weeks, followed by a second 4-week session of behavioral testing. Single-unit electrophysiology was performed within one week of the final behavioral testing session.

### Gap-prepulse inhibition of the acoustic startle (GPIAS) for tinnitus assessment

A rapid-onset sound (the startle pulse) (Fig. S1B) results in a startle response in guinea pigs, which can be reduced by presenting a prepulse stimulus (detectable above a background noise) before the startle pulse. Similarly, a background-noise gap of silence, before the startle pulse, will decrease the animal’s startle amplitude. Tinnitus that is spectrally similar to the background noise is indicated as impaired gap-detection ability^34^.

Guinea-pig startle responses were assessed by measuring their pinna-reflex displacements^33^ in response to a 20 ms startle pulse (rise-fall time: 2 ms). Pinna movements were tracked by video capture of green ink dots, manually applied to both pinnae. Offline, green pixels were identified using a custom-written k-nearest neighbors algorithm (Mathworks MATLAB)^69,70^. Frames in which green points constituted less than 0.01% of pixels were excluded, as this indicated the ears were not detectable by the camera. Pinna locations were identified by clustering green pixels and computing the centroids of a two-dimensional Gaussian mixture model fit using the Expectation–Maximization algorithm^71^. Euclidean distance between (X^ear^(t), Y^ear^(t)) points was computed over the trial duration. Trajectory accuracy was verified by trained observers. Startle amplitudes were computed by fitting the trajectory trace to a Gaussian-modulated sine-wave cycle.

Gaps in background noise (65 dB SPL; 50 ms with 5 ms rise/fall times) were presented 100 ms before a broadband noise startle pulse (90 dB SPL; 20 ms with 2 ms rise/fall times). At a given background frequency band (center frequencies of 9, 13, 17 kHz with 2 kHz bandwidths, 25 kHz with a 10 kHz bandwidth, or high pass Gaussian broadband noise), a randomized series of 10 pre-pulses (either silent gap, or a noise pre-pulse at 75 dB SPL) and 10 no-prepulse control background noises were delivered. All testing was performed in sound-proof booths (Acoustic Systems, Inc), with greater than 100 dB acoustic isolation between testing chambers. Trials were randomly presented every 20 to 30 s, with prepulse and no-prepulse trials combined into a single per-frequency testing session, and randomly interleaved. Each per-frequency testing session lasted between 9 and 10 min. Eight testing sessions were performed each testing day, for an average testing time of approximately 80 min. Guinea pigs were tested twice per week, with at least two non-testing days in between each testing day (Mondays and Thursdays or Tuesdays and Fridays) to prevent habituation. Startle amplitudes from each test session were pooled over four weeks. Startle amplitudes greater than two standard deviations above the mean were excluded from analysis. For each frequency band, a normalized startle ratio (R) was computed as the mean pre-pulse startle distribution divided by the mean of the non-pre-pulse distribution. The normalized amplitudes of the startle reflexes were compared at baseline and after noise-overexposure. An animal was defined as having tinnitus if, at a given frequency, the mean of the post-exposure distribution was significantly greater than the mean of the pre-exposure distribution (Mann–Whitney U-test; alpha = 0.05). The changes in gap R values from pre- to post-exposure were quantified by the standardized tinnitus index [(x – µ)/σ]^23,24,37^, where x is the post-exposure gap R value, µ and σ are the mean and standard deviation of pre-exposure gap R value. A significantly higher (positive) index value indicates worsened GPIAS performance and is assumed to indicate tinnitus.

### Auditory brainstem responses

All electrophysiology testing was performed in a double-walled, soundproof booth (Acoustic Systems, Inc), consistent with previous studies done in this lab^24,72,73^. Animals were anesthetized (40 mg/kg ketamine (Putney Inc.); 10 mg/kg xylazine (Lloyd Inc.)) and unilateral ABRs (Fig. S1C) were measured (tone pip, up to 1024 repetitions, 5 ms duration, 0.5 ms rise/fall time, cos^2^ gating; 8, 12, 16, 20, 24 kHz; TDT RX8 DAC, HB7 amplifier, and PA-5 attenuator). Sounds were presented closed field (DT770 Speaker) coupled to the ear canal through custom-built hollow ear bars. Calibration was performed using TDT SigCalRP, a custom MATLAB script and a ¼″ microphone (B&K 4136 and Stanford Research Systems SR760 spectrum analyzer; RX8 and PA5; 0.5 mL volume). The system transfer function was flattened with a maximum sound intensity output of 90 dB SPL using FFTs from 200 Hz-32 kHz. Stainless-steel needle electrodes were inserted into the skin overlying the bullae and at vertex. Evoked potentials were digitized (TDT RA4LI head stage; PZ2-64 pre-amp; and filtered between 300 Hz-3 kHz with a 60 Hz notch). Sounds were presented starting at 90 dB SPL and decreased in 10 dB steps to 0 dB SPL. ABR threshold for a frequency was defined as the lowest sound intensity that did not elicit ABRs with at least three identifiable peaks and troughs^73^.

### Noise overexposure

22 Guinea pigs were noise-overexposed, twice, to narrow-band noise previously shown to induce a temporary threshold shift^23,24,36,72,73^. A subset of guinea pigs (7) served as sham-exposed (anesthesia-only) controls. Guinea pigs were anesthetized with ketamine/xylazine (40:10 mg/kg). Sound-overexposures (7 kHz centered, half-octave noise at 97 dB SPL) were delivered via microphone inserts into the left ear for 2 h. ABRs were recorded before and immediately after each noise exposure, as well as prior to single-unit recordings.

### Surgical access of the cochlear nucleus

Animals were anesthetized with ketamine/xylazine (40:10 mg/kg) and placed in a hollow-ear-bar stereotaxic frame (Kopf). A custom-built heating pad with closed-loop controller was used to regulate body temperature (38 °C). Anesthetic depth was checked using a toe-pinch, and supplemental anesthesia (0.15 ketamine/xylazine dose) was administered ~ every 30 min. A craniotomy and duratomy were performed to expose the cerebellum for electrode insertion. The AVCN was accessed using previously established stereotaxic co-ordinates^44,74^.

### Single-unit electrophysiology

In vivo unit responses were recorded using multichannel recording electrodes (Neuronexus; 32 channels with 16 channels per 2 shanks; custom headstage), consistent with previous experiments in this lab^24,72,73^. Voltages were digitized (PZ2-64 pre-amp) and bandpass filtered (300 Hz-3 kHz, with a 60 Hz and harmonic comb-filter). Spike thresholds were identified when voltage amplitude crossed 2 standard deviations above the mean voltage arising from spontaneous activity (see Fig. 3C for example threshold-triggered mean waveforms). Units were identified by their responses to 65 dB SPL wideband (200 Hz-40 kHz) search stimuli. Neuron thresholds were stable throughout the experiment with thresholds varying between 40- and 50-dB SPL. Neural spike data was imported into MATLAB and analyzed offline. Spike waveforms were projected into principle component (PC) space and manually clustered by the first three coefficients by a trained user. Unit consistency was maintained by clustering all PC coefficients from a given recording location throughout the experiment. Timestamps were grouped by cluster into isolated units, and spiketrains constructed in MATLAB. Putative bushy-cell single units were identified by their receptive fields (10 dB steps from 0 to 90 dB SPL; frequencies logarithmically spaced from 2 to 24 kHz in 0.25 octave steps) (colormap in Fig. S1D), and either primary-like or primary-like-with-notch peri-stimulus time histograms (PSTH) (lower inset of Fig. S1D)^75–77^. Bushy cells can sometimes be identified by the presence of a pre-potential, arising from the large, tightly coupled auditory nerve endbulb of Held input on the bushy-cell soma^10,59,78^. Using silicon substrate electrodes, prepotentials can sometimes be identified on putative bushy cells due to low signal-to-noise ratios, as shown previously^74^. Thus, in a subset of units, pre-potentials were identified (upper inset in Fig. S1D). Once putative bushy cells were identified, spontaneous activity was collected (at least 150 s) followed by unit responses to BF tones and broadband noise over a range of intensities (5 dB steps from 0 to 90 dBSPL) (rate-intensity function: filled symbols in Fig. S1E). 1111 putative bushy cells were identified for analysis, and non-bushy cells were excluded from further analysis. First-spike latency (FSL) was assessed by recording the first spike timestamp post-stimulus onset for each trial (n = 100 trials) during rate-intensity function recordings, and the mean for all trials computed (latency-intensity function: open symbols in Fig. S1E). FSL jitter was assessed as the standard deviation of the FSL distribution.

### Monotonicity fraction

RIFs non-monotonicity was quantifed^32^ by computing the Non-Monotonicity Fraction, which is calculated as the percent of slope decreases over the sound intensity range used to elicit the RIF above threshold. A unit was defined as non-monotonic if the non-monotonicity fraction exceeded 12.5% of the RIF (BF-tone for Fig. S2A, BBN-noise RIFs for Fig. S2B).

### Hyperacusis Index

A Hyperacusis Index (HI) was computed for each unit as the geometric mean of the unit’s 1) average rate-intensity-function slope at intensities greater than 40 dB SPL (nearest base-10 multiple of the average population threshold; 42.1 ± 0.48 dB SPL) and less than 90 dB SPL (the maximum calibrated system output for all tested frequencies) and 2) peak firing rate at best frequency from 40 dB SPL to 90 dB SPL.

$$ HI = \surd \left( { RIF\,Slope*Max\,\left( {Firing\,Rate} \right)} \right) $$

### Synchrony and spontaneous firing rate assessments

Cross-unit synchrony was computed using cross-correlograms for evoked^43^ and spontaneous activity^23,24,38^. For spontaneous synchrony, 150 s of spontaneous activity was recorded. SFR was computed as the average spike rate during this trial. For spontaneous synchrony calculations, spikes co-occurring within a 150 µs window were removed. For evoked activity, spikes from frequency-intensity stimulus pairs between receptive fields were pooled. Cross-correlation coefficients (*p*(τ)) were computed as a function of time lag for each pairwise combination of spike trains.

$$ p\left( \tau \right) = \frac{{R_{AB} \left( \tau \right) - E}}{{\sqrt {N_{A} N_{B} } }}, E = \frac{{N_{A} N_{B} }}{n} $$

$R_{AB} \left( \tau \right)$ is the unbiased cross-correlation of spike trains *A* and *B*; *N*_*A*_ and *N*_*B*_ indicate spike counts in the respective spike trains. *E* is the mean probability of coincident firing for Poisson-distributed data, defined by the multiplication of *N*_*A*_ and *N*_*B*_ over the number of bins (*n*). Bin size was constant at 0.3 ms^43^. A unit-pair was considered synchronous when the peak *p* value was greater than ± 4 standard deviations from the mean *p*(τ). In the present study, negative cross-correlations were removed from further analysis.

### Statistical analysis

Correlation coefficients were computed using Pearson’s algorithm. Gaussian-mixture models were fit using the Expectation–Maximization algorithm (MATLAM *fitgmdist*). Distribution differences were assessed for significance with ANOVAs, Kruskal–Wallis or two-sample KS tests where appropriate (alpha = 0.05). Post-hoc corrections for multiple comparisons were done using the Bonferroni method. The experimenter was blinded as to the status of each animal regarding exposure of behavioral outcome.

### Dorsal cochlear nucleus fusiform cell data and analyses

Data published in Wu et al.^24^ were further analyzed in this study with the permission of the authors. Tinnitus behavioral status in all animals was determined following the GPIAS paradigm presented earlier. SFR, spontaneous synchrony and HI analyses were performed identically between DCN and VCN data. Data were normalized by the control-animal SFR maximum where indicated.

## Supplementary information

Supplementary Information 1.

Supplementary Information 2.
