# Supplementary material for: Ventral cochlear nucleus bushy cells encode hyperacusis in guinea pigs
Source: Sci Rep. 2020 Nov 26;10:20594. doi: 10.1038/s41598-020-77754-z (PMC7693270; doi:10.1038/s41598-020-77754-z)
Supplement: Supplementary file 1 — Supplementary Information 1. [file 41598_2020_77754_MOESM1_ESM.docx]

**Supplemental Figures.**

1. **Experimental paradigm.** **A)** Animals underwent four weeks of baseline behavioral testing (time = 0 weeks to t = 4 weeks), followed by two separate noise-exposures, each four weeks apart (t(1,2) = 4, 8 weeks). Auditory brainstem responses (ABRs) were measured before and immediately after each noise-exposure. After another four weeks, behavioral testing was resumed (t = 12 weeks) for four weeks, after which electrophysiology experiments were performed (t = 16 weeks). **B)** Guinea pigs startle in response to loud, unexpected sounds (blue pulse embedded in black carrier band; top half of panel), and startle less when a gap in the background noise precedes the loud sound (bottom half of panel). This phenomenon is termed gap-prepulse inhibition of the acoustic startle (GPIAS) and is reduced in animals with tinnitus. **C)** Representative ABR waveform with symbols indicating wave 1 peak and trough (teal stars) and measured W1 amplitude. **D)** Sample receptive field (colormap; cool color reflects low firing rates while warm reflects high firing rates), primary-like peri-stimulus time histogram (PSTH; bottom inset) and spike with prepotential (top inset) from a putative bushy cell. **E)** Rate- and latency-intensity functions (RIF: filled symbols, left axis; LIF: open symbols, right axis), from a putative bushy cell, were measured in response to the best-frequency tones (BF; black) and to broadband noise (orange).
2. **Noise-exposure increases monotonicity of RIFs and creates population of *Hyperacusis Units*.** A unit RIF was defined as non-monotonic if the non-monotonicity fraction was greater than 12.5% (dashed, vertical orange line). **A)** The distribution of RIF non-monotonicity from bushy cells in noise-exposed animals (purple bars) and controls (black bars) arising from tones at unit BF. **B)** RIF non-monotonicity distribution for BBN RIFs. **C)** Probability (Gaussian-mixture model) of classifying a bushy cell as a Hyperacusis Unit, with Pr(Hyperacusis) greater than or equal to 0.5 (dashed vertical line) for hyperacusis units and less than 0.5 for non-Hyperacusis Units. The HI-value at Pr=0.5 point is 35.5, which served as the Hyperacusis Threshold.
