# Supplementary material for: Ventral cochlear nucleus bushy cells encode hyperacusis in guinea pigs
Source: Sci Rep. 2020 Nov 26;10:20594. doi: 10.1038/s41598-020-77754-z (PMC7693270; doi:10.1038/s41598-020-77754-z)

Title: Ventral cochlear nucleus bushy cells encode hyperacusis in guinea pigs.

Abbreviated title: Neural contributors to hyperacusis

Authors: David T. Martel[1,2] and Susan E. Shore *[1,2,3]

Departments of [1] Otolaryngology, [2] Biomedical Engineering, [3] Department of Molecular and Integrative Physiology, University of Michigan, Ann Arbor, 48109

Author of Correspondence: Susan E. Shore

Kresge Hearing Research Inst. 1100 W. Medical Center Drive, Ann Arbor, MI 48104, USA.

Tel: 734-647-2116; Fax: 734-764-0014; Email: sushore@umich.edu

Number of pages. 25

Number of figures, tables, multimedia and 3D models. 6 + 2 supplemental

Number of words:

Abstract: 179, **Introduction:** 488, **Results:** 2032, **Discussion:** 1251, **Methods:** 1855

Competing Interests

The authors declare no competing financial interests.

Acknowledgements

This work was supported by National Institutes of Health Grants R01-DC004825 (SES), RF1-MH114244-01 (SES), T32-DC00011 (DTM) and P30-DC05188. We thank Calvin Wu and Michael Roberts for technical and analytical assistance and review of an earlier version of this manuscript, Adam Hockley and Mike Selesko for behavioral data collection and data analysis assistance, and Mr. Deepak Dileepkumar for technical support.

Figure S1.

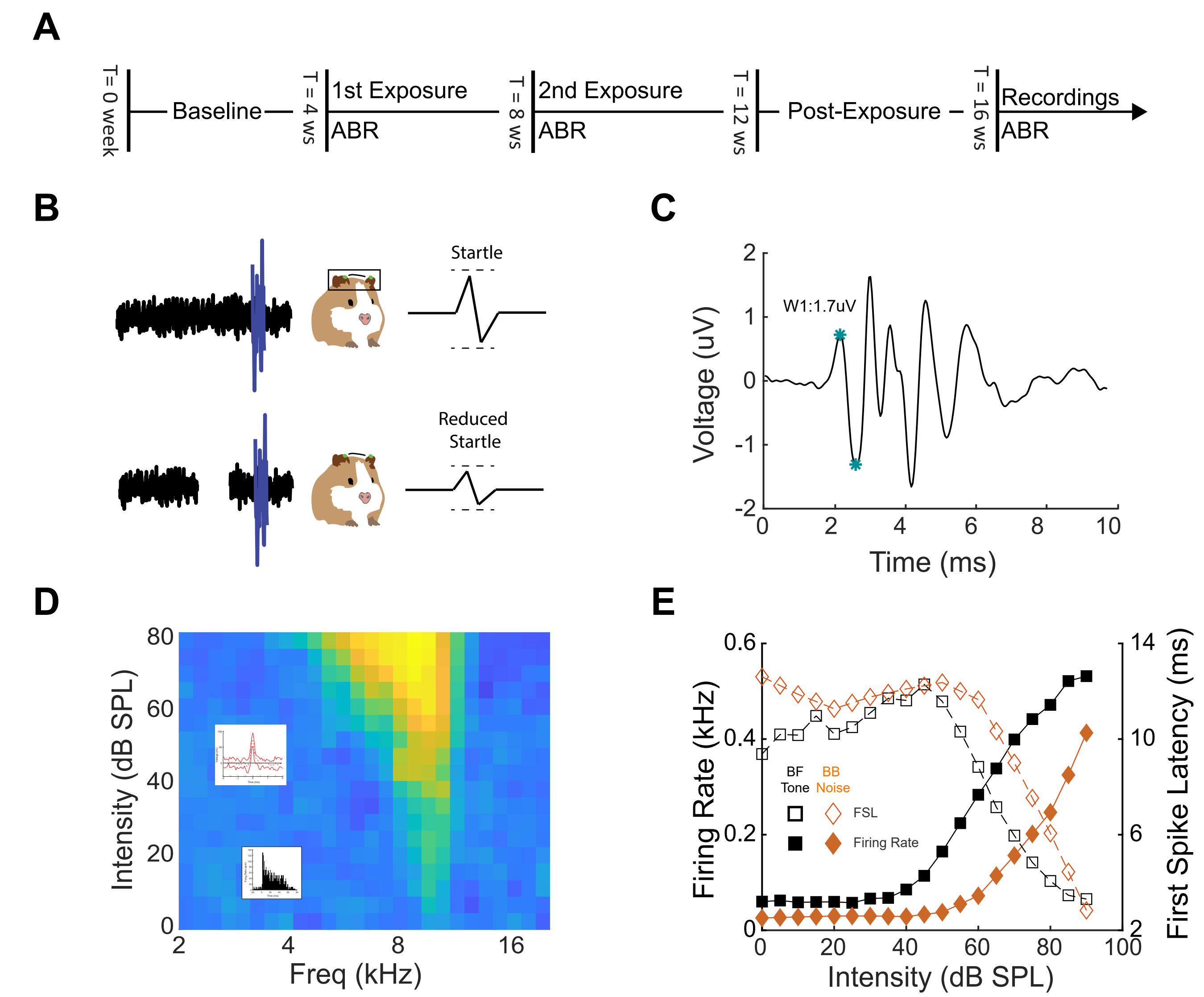

Figure S2.

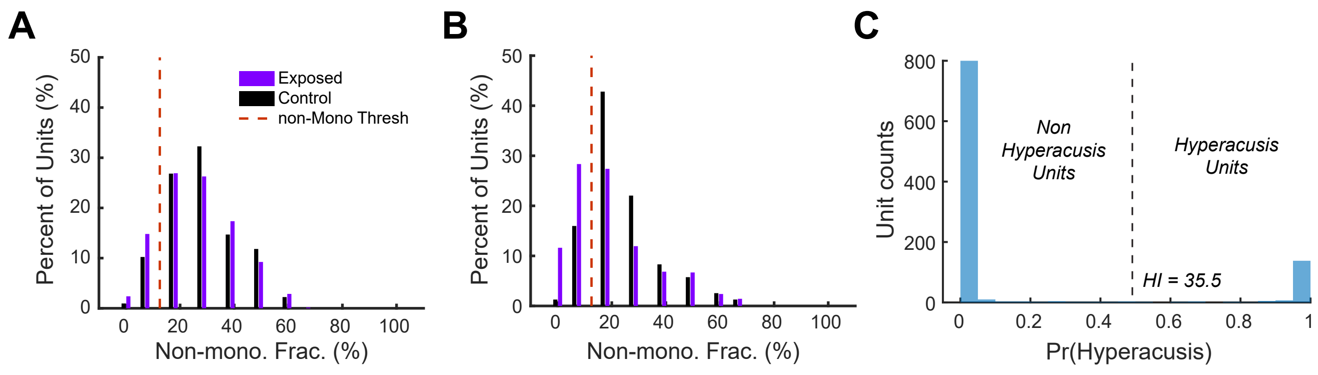

Supplement: Supplementary file 2 — Supplementary Information 2. [file 41598_2020_77754_MOESM2_ESM.pdf]
